# A paradigm shift in diabetes care: clinical implications of once-weekly insulin icodec

**DOI:** 10.1097/MS9.0000000000005132

**Published:** 2026-05-12

**Authors:** Muhammad Ali Abid, Muhammad Hafi Abid, Muhammad Daniyal Afzal

**Affiliations:** a Department of Medicine, King Edward Medical University, Lahore, Pakistan; b Department of Medicine, Faisalabad Medical University, Faisalabad, Pakistan; c Department of Medicine, Kyrgyz State Medical Academy, Bishkek, Kyrgyzstan

**Keywords:** insulin icodec, diabetes mellitus, insulin, treatment adherence, FDA approval

## Abstract

Diabetes mellitus is a metabolic disorder characterized by hyperglycemia, which occurs due to problems in insulin secretion, insulin action, or both, and remains a major global health burden. Insulin therapy is central to the management of type 1 diabetes and is frequently required in advanced type 2 diabetes, with conventional regimens involving multiple daily injections that may negatively impact adherence. Awiqli™ (insulin icodec-abae), a novel once-weekly basal insulin analog, has recently been approved to improve glycemic control in adults with type 2 diabetes. Its mechanism involves reversible albumin binding, forming a circulating depot that enables a prolonged half-life of approximately 1 week and provides stable glucose-lowering effects. Evidence from multiple randomized, treat-to-target clinical trials demonstrates that once-weekly insulin icodec achieves glycemic control comparable to, and in some cases superior to, once-daily basal insulin analogs such as insulin glargine and insulin degludec. The most commonly reported adverse effects include hypoglycemia, injection site reactions, and weight gain. The reduced injection frequency offers a significant advantage in improving treatment convenience and adherence; however, its prolonged duration of action necessitates cautious dose titration and limits flexibility during acute clinical changes. Overall, insulin icodec represents an important advancement in diabetes care, with the potential to simplify insulin regimens and improve patient-centered outcomes, although further studies are warranted to evaluate long-term safety and broader clinical applications.

## Introduction

Diabetes is a chronic disease characterized by elevated levels of glucose in the blood resulting from abnormalities in β-cell function that impair insulin action, insulin resistance, or both^[^1^]^. The two most common forms of diabetes are type 1 diabetes and type 2 diabetes^[^1^]^. Type 1 diabetes commonly develops in childhood, whereas type 2 has a strong genetic predisposition and is closely related to obesity and physical inactivity^[^1^]^. Type 1 diabetes is characterized by inadequate insulin production and therefore requires lifelong insulin therapy^[^2^]^. The exact cause of the disease is uncertain, and there are no established methods for its prevention^[^2^]^. Type 2 diabetes occurs as a result of insulin resistance, meaning the body is unable to use insulin effectively, which results in high blood sugar levels^[^2^]^. Type 2 diabetes is often preventable by managing modifiable risk factors such as weight and physical activity^[^2^]^. If left untreated, it can lead to damage to nerves and blood vessels over time, making early detection and regular checkups key components of management^[^2^]^.

## Epidemiology and global burden of diabetes

In 2022, about 14% of adults aged 18 years and older had diabetes, while over half (59%) of adults aged 30 years and above were not on any medication for their condition^[^3^]^. In 2021, diabetes was directly responsible for 1.6 million deaths, with 47% of these deaths occurring before the age of 70 years^[^3^]^.

## Role of insulin therapy

Insulin is the hormone responsible for glucose metabolism^[^4^]^. It lowers blood glucose by enhancing glucose uptake in peripheral tissues, particularly skeletal muscle and adipose tissue, and by reducing glucose production by the liver^[^4,5^]^. Insulin additionally inhibits lipolysis and proteolysis while stimulating protein synthesis^[^4,5^]^. Insulin is the mainstay of management for type 1 diabetes, while type 2 diabetes can be managed with insulin injections or other medicines^[^6,7^]^. Patients with type 1 diabetes always require both basal and bolus insulin, while patients with type 2 diabetes who need insulin usually start on basal insulin, with prandial insulin added in case of suboptimal blood glucose control^[^7^]^.

Basal insulin options consist of intermediate-acting, long-acting, and ultra-long-acting insulin formulations^[^7^]^. Intermediate-acting insulin, neutral protamine Hagedorn (NPH), has a duration of action of approximately 12 hours, with a peak effect seen at 4–6 hours^[^7^]^. Compared with NPH, long-acting and ultra–long-acting insulins are absorbed more slowly and do not produce a peak, providing a more stable basal coverage^[^7^]^. Ultra-long-acting insulins have a duration of action longer than 24 hours, allowing for once-daily administration^[^7^]^. Ultra–long-acting insulins provide stable glycemic control and a comparable or reduced risk of hypoglycemia in both type 1 and type 2 diabetes^[^7^]^.

Bolus options, used to provide postprandial control, consist of short-acting, rapid-acting, and ultra-rapid-acting insulins^[^7^]^. Short-acting insulin includes regular insulin, which has an onset of action of 30–60 minutes, a duration of 5–8 hours, and a peak concentration at 80–120 minutes after injection^[^7^]^. Rapid-acting insulins include insulin aspart, lispro, and inhaled insulin^[^7^]^. These act faster than regular insulin, with an onset around 10–20 minutes, a duration of 3–5 hours (35–55 minutes for inhaled insulin), and a peak at 30–90 minutes (inhaled insulin peaks at 35–55 minutes)^[^7^]^. Ultra-rapid-acting insulins, such as faster aspart and lispro-aabc, have an even faster onset of action^[^7^]^. Faster aspart has an onset at 16–20 minutes, a duration of 5–7 hours, and peaks at about 63 minutes^[^7^]^. Lispro-aabc has an onset at 15–17 minutes, a duration of 4.7–7.3 hours, and peaks at about 57 minutes^[^7^]^. Mixed insulin products contain combinations of varying concentrations of both basal and bolus coverage in the same injection^[^7^]^.

## Once-weekly basal insulin: insulin icodec

Multiple daily injections and injection site reactions represent a major inconvenience for patients^[^8^]^. The US Food and Drug Administration (FDA) has recently approved Awiqli^TM^ (insulin icodec-abae), a once-weekly subcutaneous injection, to improve glycemic control in adults with type 2 diabetes^[^4^]^. The FlexTouch pen delivers insulin in 10-unit increments and allows delivery of up to 700 units in a single injection, where one unit corresponds to one international unit of human insulin^[^4^]^. Awiqli^TM^ binds reversibly to albumin, forming a circulating depot from which insulin icodec-abae is gradually released^[^4^]^. This sustained release activates insulin receptors and provides a stable glucose-lowering effect^[^4^]^. When insulin icodec-abae binds to insulin receptors, it produces pharmacologic effects comparable to those of human insulin^[^4^]^. Following subcutaneous administration, insulin icodec-abae has an approximate half-life of about 1 week, which remains consistent across different dose levels^[^4^]^. Its degradation is similar to that of human insulin, and all metabolites formed are inactive^[^4^]^.

## Clinical trial evidence supporting approval

The approval by the FDA was based on results from three randomized, open-label, treat-to-target, active-controlled trials and one randomized, double-blind, treat-to-target, active-controlled trial. In these studies, Awiqli^TM^ (insulin icodec-abae) was used either with mealtime insulin or alongside common oral antidiabetic agents and/or GLP-1 receptor agonists^[^4^]^. Blinded continuous glucose monitoring was employed in Trials A, C, and D. Across all trials, insulin icodec-abae doses were adjusted in 20-unit increments, and a target fasting plasma glucose (FPG) level of 80–130 mg/dL, calculated from the mean of the three most recent FPG readings, was used^[^4^]^.

In Trial A (NCT04460885), 984 participants were randomized to receive either once-weekly Awiqli^TM^ (insulin icodec-abae) or once-daily insulin glargine U-100 according to approved dosing guidelines^[^9^]^. Preexisting non-insulin antidiabetic therapies were continued in both groups throughout the trial, except for glinides and sulfonylureas, which were discontinued at randomization^[^9^]^. The study population included insulin-naïve adults with type 2 diabetes inadequately controlled with one or more oral antidiabetic drugs (OADs) or GLP-1 agonists^[^9^]^. The study population had a mean age of 59 years, an average diabetes duration of 12 years, and a mean body mass index (BMI) of 30.1 kg/m^2[^9^]^. After 52 weeks, once-weekly Awiqli^TM^ caused a significant reduction in HbA1c compared with once-daily insulin glargine U-100, with an estimated treatment difference of −0.18 [95% confidence interval (CI): −0.29 to −0.08], and showed an increase in time in range (TIR) by 4.27%^[^4,9^]^.

In Trial B (NCT04795531), 588 participants were randomized to receive either once-weekly Awiqli^TM^ or once-daily insulin degludec U-100 according to approved dosing guidelines^[^10^]^. Preexisting non-insulin antidiabetic therapies were continued in both groups throughout the study, except for sulfonylureas and glinides, which were reduced by approximately 50% at randomization at the investigator’s discretion^[^10^]^. The study population had a mean age of 58 years, an average diabetes duration of 11 years, and a mean BMI of 29.6 kg/m^2[^10^]^. After 26 weeks, once-weekly Awiqli^TM^ produced a statistically significant reduction in HbA1c compared with once-daily insulin degludec U-100 with an estimated treatment difference of −0.22 (95% CI: −0.35 to −0.09)^[^4,10^]^.

In Trial C (NCT04770532), 526 participants were randomized to receive either once-weekly Awiqli^TM^ (insulin icodec-abae) or once-daily insulin degludec U-100 according to approved dosing guidelines^[^11^]^. Preexisting non-insulin OADs or GLP-1 receptor agonists were continued in both groups throughout the trial, except for glinides and sulfonylureas, which were discontinued at randomization^[^11^]^. The study population had a mean age of 62 years, an average diabetes duration of 17 years, and a mean BMI of 29.6 kg/m^2[^11^]^. After 26 weeks, once-weekly Awiqli^TM^ demonstrated a significant decrease in HbA1c compared with once-daily insulin degludec U-100, with an estimated treatment difference of −0.19 (95% CI: −0.32 to −0.06)^[^4,11^]^.

In Trial D (NCT04880850), an open-label, active-controlled, parallel-group, multicenter, multinational, treat-to-target trial, 582 patients were randomized to receive either once-weekly Awiqli^TM^ or once-daily insulin glargine U-100, both administered with insulin aspart before meals according to approved labeling^[^12^]^. Preexisting non-insulin OADs or GLP-1 receptor agonists were continued in both groups throughout the trial, except for glinides and sulfonylureas, which were discontinued at randomization^[^12^]^. The study population had a mean age of 60 years, an average diabetes duration of 17 years, and a mean BMI of 30.3 kg/m^2[^12^]^. After 26 weeks on once-weekly Awiqli^TM^, HbA1c had decreased significantly compared with once-daily insulin glargine U-100, with an estimated treatment difference of −0.02 (95% CI: −0.11 to −0.15)^[^4,12^]^. TIR over weeks 22–26 was similar between groups: 66.76% in the Awiqli^TM^ arm and 66.46% in the insulin glargine U-100 arm^[^4,12^]^.

## Efficacy and safety profile

In insulin-naïve and basal-only patients with type 2 diabetes, Awiqli^TM^ produced a statistically significant improvement in glycemic control compared with insulin glargine U-100 or insulin degludec U-100^[^4^]^. Among patients already on a basal-bolus regimen, Awiqli^TM^ achieved glycemic control comparable to that of insulin glargine U-100 or insulin degludec U-100^[^4^]^. The adverse reactions associated with Awiqli^TM^ were hypoglycemia, injection site reactions, lipodystrophy, hypersensitivity reactions, pruritus, rash, edema, and weight gain^[^4^]^. Due to the prolonged half-life of Awiqli^TM^, dose modification is generally not recommended during acute illness or in response to short-term changes in physical activity or dietary intake^[^4^]^. In such situations, alternative strategies should be used instead, such as adjusting carbohydrate intake or modifying other glucose-lowering medications^[^4^]^.

## Clinical implications and advantages

The approval of once-weekly Awiqli^TM^ (insulin icodec-abae) marks a significant milestone in diabetes care, especially for type 2 diabetes, by substantially reducing the injection burden associated with daily basal insulin therapy and potentially enhancing long-term adherence and overall glycemic consistency. Its importance is highlighted by evidence showing that once-weekly dosing can provide basal insulin coverage comparable in efficacy to conventional daily insulin analogs, while also improving patient convenience and reducing injection fatigue, thereby positively influencing quality of life.

## Limitations and future directions

However, several limitations remain, including the requirement for cautious dose titration due to its ultra-long half-life, which limits rapid dose adjustments during acute clinical changes such as acute illness, surgery, or rapidly changing insulin needs. The prolonged duration also raises concern for delayed or prolonged hypoglycemia if overdosed, and there is less room for rapid correction compared to shorter-acting basal insulins. Moreover, its application is currently confined to basal insulin replacement, and it does not address postprandial glucose control in advanced diabetes. Future research is expected to focus on refining individualized dosing strategies, assessing long-term cardiovascular and renal outcomes, investigating fixed combinations with agents such as GLP-1 receptor agonists, and expanding its clinical use to earlier stages of type 2 diabetes and possibly selected type 1 diabetes populations, where improved adherence and simplified regimens could significantly enhance treatment outcomes.

## Conclusion

Awiqli™ (insulin icodec-abae) represents a meaningful advancement in basal insulin therapy for type 2 diabetes by enabling once-weekly dosing, thereby reducing injection burden and potentially improving adherence. Clinical trials demonstrate glycemic control comparable to, and occasionally better than, daily basal insulin analogs, with an acceptable safety profile. However, its ultra-long half-life limits flexibility in dose adjustments during acute clinical changes and confines its role to basal insulin replacement. Overall, insulin icodec offers a promising, patient-centered approach to simplifying diabetes management, with further research needed to optimize its long-term use and clinical outcomes.

## Data Availability

Not applicable to this study.

## Research registration unique identifying number (UIN)

Not applicable to this study.
